# Where does endogenous oxalate come from? - a systematic review of endogenous sources of urinary oxalate

**DOI:** 10.1007/s00240-026-01995-2

**Published:** 2026-04-30

**Authors:** Brian Dick, Jorge Mena, Marshall Stoller

**Affiliations:** Department of Urology, University of California, San Francisco, San Francisco, CA USA

**Keywords:** Oxalate, Urine, Metabolism, Endogenous, Quantification

## Abstract

Oxalate is a toxic waste product in humans, capable of complexing with calcium to form kidney stones. While some oxalate is consumed in the diet, there are endogenous sources in humans. This review compiles known endogenous sources of urinary oxalate. PubMed and Embase databases were searched, as well as backward citations, for relevant articles published in English. The search strategy relied on terms related to endogenous synthesis and metabolic tracing. Title/abstract review was performed, and articles were excluded from full review if they did not report data on human sources of endogenous oxalate. Risk of bias was assessed using a modified JBI checklist. Weighted averaging was used to combine results and predict endogenous oxalate production. Of 228 unique articles identified, 23 were eligible and included in this study. Total endogenous oxalate production was reported by 7 of the papers, representing 305 healthy subjects. Weighted mean of endogenous oxalate production was 23.8 mg/day (95% CI 23–24.6 mg/day) with a range of 17–34 mg/day. Daily turnover from the metabolic pool of ascorbic acid may account for up to 40% − 60% of total endogenous oxalate production. Other contributors include hydroxyproline (~ 15%), glycine (< 10%), and glycolate (~ 4%). Phenylalanine (< 0.7%), and fructose (negligible) contribute minimally. This review highlights that daily turnover from the metabolic pool of ascorbic acid is the primary source of endogenous oxalate. However, current estimates rely on highly variable data dating back nearly 70 years. Future research using modern metabolomics is required to re-characterize this pathway.

**Supplementary Information:**

The online version contains supplementary material available at 10.1007/s00240-026-01995-2.

## Introduction

Nephrolithiasis is a highly prevalent condition, affecting 1 in 10 American adults [1]. Calcium oxalate stones are the most common and make up nearly 60% of all calculi [2]. Large cohort studies evaluating 24-hour urine chemistries have shown that lower levels of absolute urinary oxalate are independently associated with a decreased relative risk of stones, making oxalate a target of medical management [3]. Total urinary oxalate comes from exogenous consumption (i.e. through diet) and endogenous production. In healthy individuals, each of these contribute in approximately equal proportions [4].

Current guidelines from the American Urological Association recommend that calcium oxalate stone formers restrict their dietary oxalate [5]. However, there are no clinical guidelines for managing endogenous oxalate production. Various metabolic pathways of endogenous oxalate production have been investigated using dietary studies and isotope infusions, but the literature remains fragmented. Additionally, past research has been centered on patients with genetic disorders of oxalate handling, like the primary hyperoxalurias.

This paper aims to review the existing literature and identify all known sources of endogenous oxalate in healthy individuals, without known genetic disorders of oxalate handling. A clearer understanding of these pathways is essential for identifying new therapeutic targets for the prevention of stone recurrence.

## Methods

### Search strategy

On December 15th, 2025, PubMed and Embase databases were searched to identify articles relating to endogenous oxalate production. Backwards citation searching was permitted for articles that were reviewed in full. Inclusion criteria included publication in English language and quantification of urinary oxalate. Articles were excluded if they did not involve human participants, if they exclusively included participants who had altered oxalate production/ excretion (primary hyperoxaluria, chronic kidney disease), or if they did not report *endogenous* oxalate production. This study protocol was not prospectively registered. To minimize the risk of bias, the inclusion criteria and search strategy were established prior to data extraction.

The search string relied on 4 key elements: (1) broadly identifying all articles pertaining to oxalate; (2) filtering for articles also pertaining to urine; (3) filtering for titles and abstracts that included words pertaining to synthesis, endogenous production, or precursors of oxalate; and (4) filtering for articles that used quantitative measurement tools. The PubMed and Embase search strings are shown in the supplementary files as S1.

### Article review

A single author (BD) screened titles and abstracts to identify articles that may quantify endogenous sources of oxalate production. If there was uncertainty, the article was included for full review. A second author (JM) performed a confirmatory screen of the title and abstract of all excluded articles. If either author deemed an article eligible for inclusion, it was included in full-article review. One author (BD) then screened the full-text articles for inclusion. A flow diagram was generated using online software [6].

### Bias assessment

To minimize risk of bias, a custom checklist adapted from JBI critical appraisal tools was developed and applied to the studies. Items that did not apply to metabolic studies (i.e. “Did the case series have consecutive inclusion of patients?”) were removed and questions about methodology (i.e. “Did the study control for exogenous oxalate?”) were added. This checklist can be found in the supplementary files as S2. One author (BD) applied the tool to studies being considered for data extraction and analysis. The assessment tool was visualized using a stoplight system – green indicates that a domain requirement was explicitly met, yellow indicates unclear or insufficient data (i.e. participants were labeled “healthy” but there was no BMI data); red indicates a failed domain (i.e. inclusion of known stone formers). Domains 3, 5, and 6 were categorized as critical – they respectively represent control for dietary oxalate, acidification of collected urine, and oxalate quantification assay. Failure in any of these domains resulted in study exclusion from pooled analysis due to high-risk of bias. Studies that failed non-critical domains were included in analysis but classified as having a medium risk of bias.

### Data extraction and analysis

The primary outcome was proportional contribution of oxalate precursors to total endogenous oxalate production. The secondary outcome was total endogenous oxalate production. Additionally, data on sample size, patient comorbidities, and methodology (dietary restrictions, oxalate quantification method, urine handling, oxalate assay) were extracted.

Studies were considered eligible for data synthesis if they quantified the total amount of oxalate excreted in a day or reported the contribution of metabolic precursors to total oxalate production. All urinary oxalate measurements were converted to milligrams per day (mg/ day). When values were reported in mmol, standard molecular weights were used for conversion. The contribution of specific metabolic precursors to overall oxalate production were expressed as percentage of total production. Total oxalate production was reported as a weighted mean with 95% confidence interval.

## Results

**Fig. 1:**
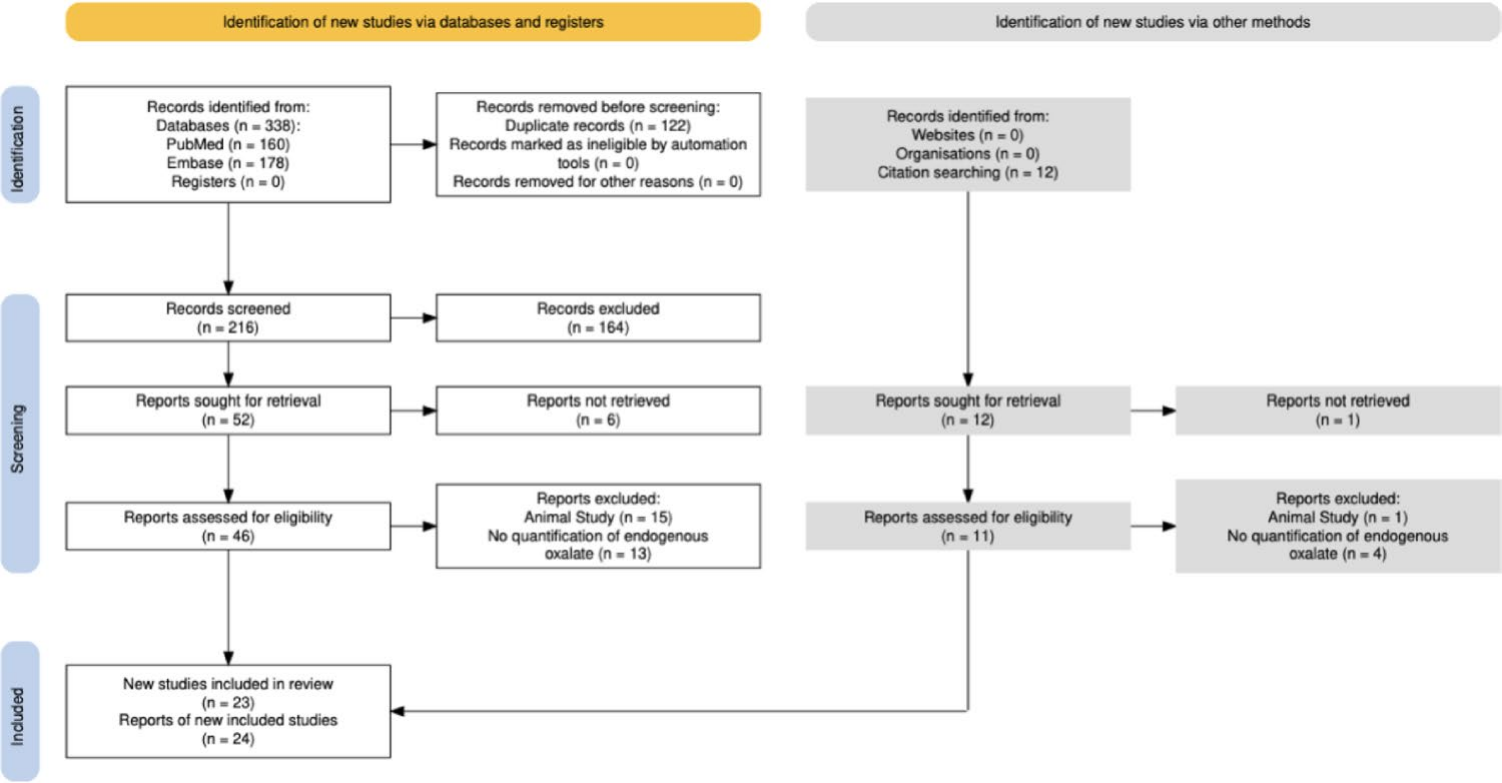
PRISMA flow diagram

338 records were identified from PubMed and Embase. After duplicate removal, 216 records were screened, from which 46 full text documents were reviewed. An additional 12 records were identified via backwards citation searching of reviewed records. In total, 23 studies were included for bias assessment and data extraction [7–29]. The search selection process is shown as a flow diagram in Fig. 1.

### Risk of bias

A custom, JBI style checklist was used to assess risk of bias as described in the methods. A summary of the assessment is shown as a stoplight chart in Table 1. Around half of the studies were determined to have a high risk of bias and were excluded from pooled analysis. Of the studies included in data analysis, eight had a medium risk of bias and only four had a low risk of bias.

**Table 1:**
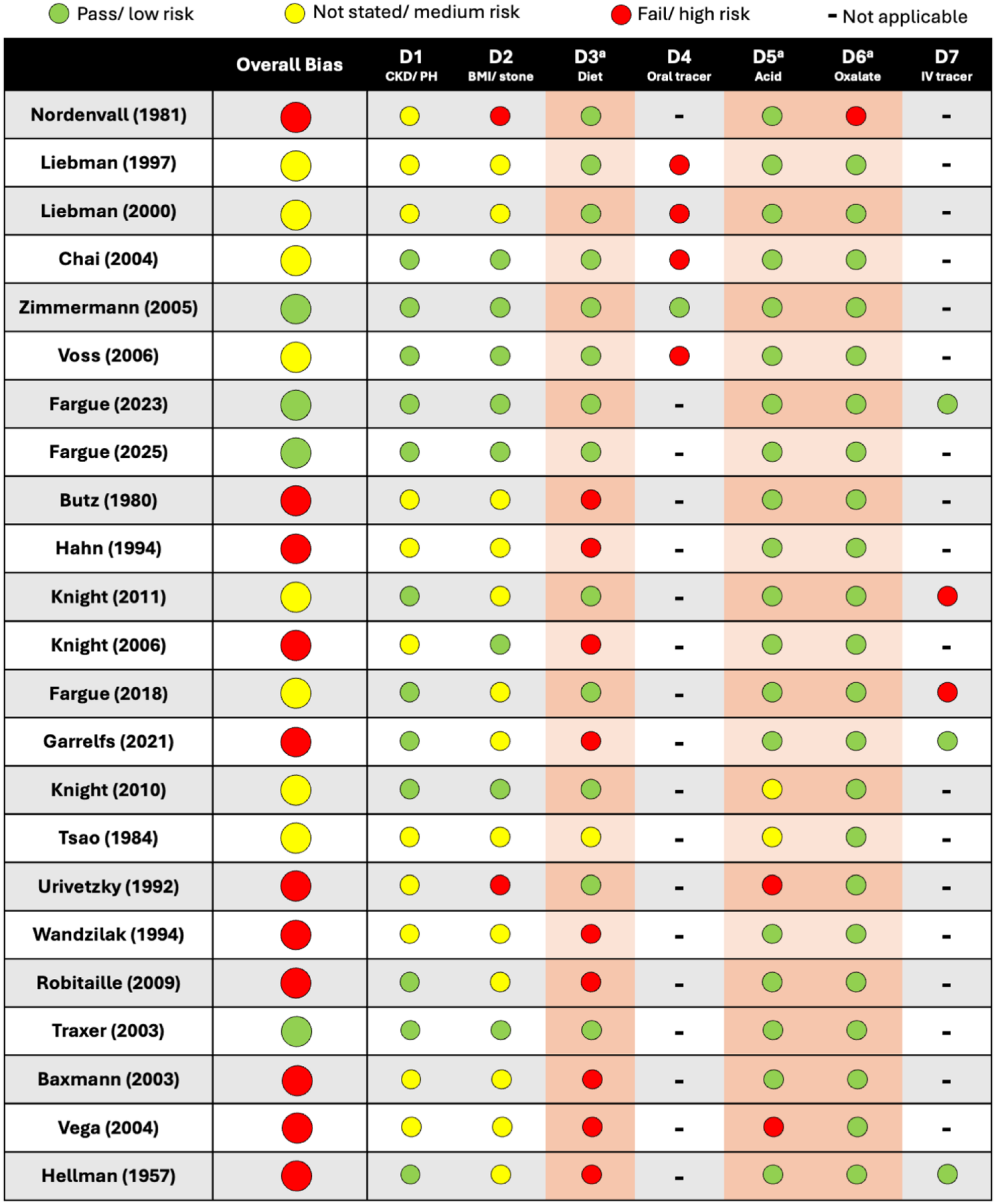
Stoplight chart showing bias assessment of relevant studies

D1 – Does the study involve participants that were free of genetic or renal defects that alter oxalate handling (i.e. CKD or primary hyperoxaluria (PH))?

D2 – Does the study outline patient demographics possibly related to endogenous oxalate synthesis (i.e. BMI, stone formers and non-stone formers)?

D3 – Does the study control for diet / exogenous oxalate consumption?

D4 – If an oral tracer was used, did the study account for the higher bioavailability of the tracer relative to dietary oxalate?

D5 – Is urine acidified upon collection to prevent non-enzymatic conversion of ascorbic acid to oxalate?

D6 – Was the oxalate assayed in a reliable way for all participants?

D7 – For tracer studies, was steady state reached?

^a^ Critical domain. Failure = high risk of overall bias

### Total endogenous oxalate production

Nine studies reported total endogenous oxalate production and are summarized in Table 2. Two of those were excluded from pooled analysis due to high risk of bias - Nordenvall et al. [7] used an outdated oxalate assay with poor specificity while Garrelfs [20] et al. did not control for exogenous oxalate consumption. The remaining 7 studies represent 305 patients and used various methods to identify endogenous production. Most of them calculated endogenous production by subtracting tracer-measured exogenous absorption from total excretion. Fargue et al. [14] placed participants on a low-oxalate diet followed by overnight fast, a method they proved effective in their 2023 paper [13]. The weighted mean endogenous oxalate production was 23.8 mg/day (95% CI 23–24.6 mg/day).

**Table 2:**
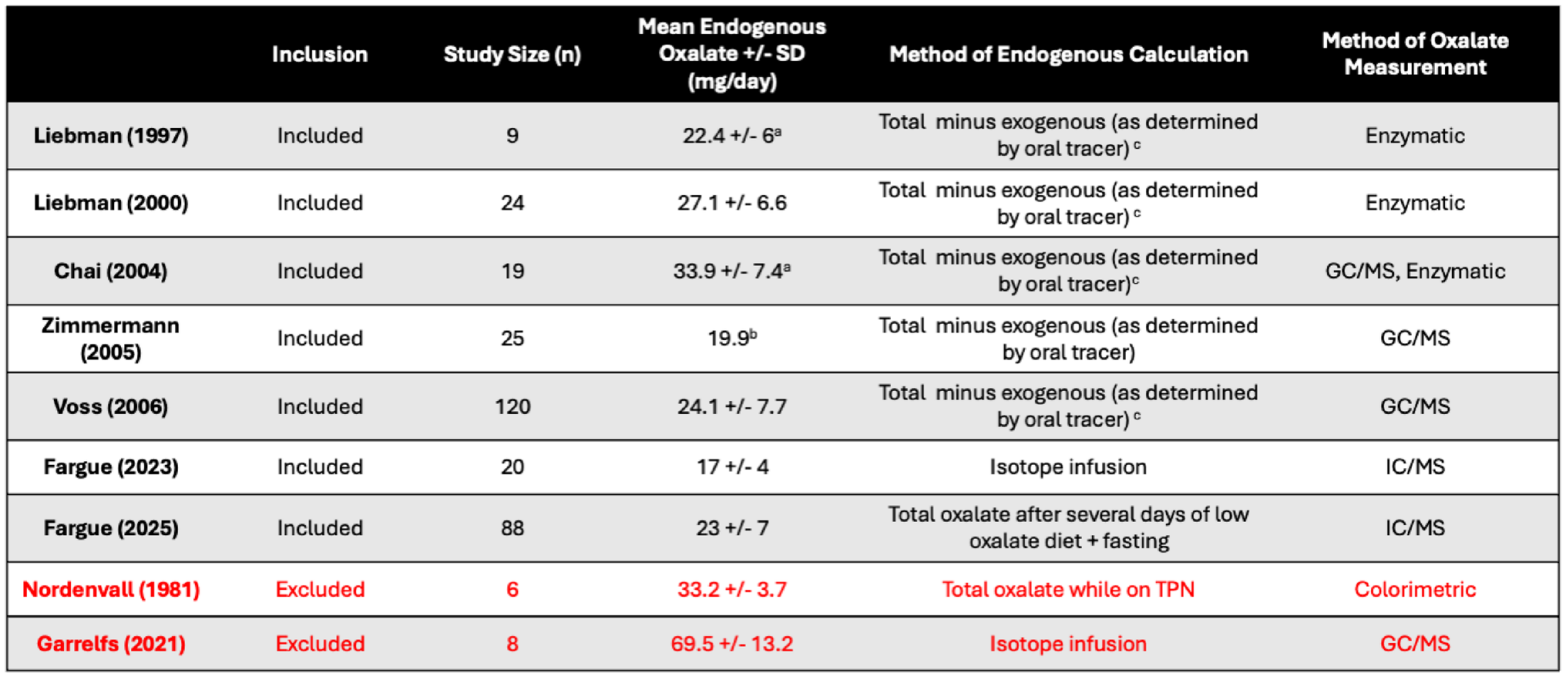
Studies reporting total endogenous oxalate synthesis in healthy subjects. GC/MS = gas chromatography and mass spectroscopy, IC/MS = ion chromatography and mass spectroscopy

^a^ Standard deviation calculated from SEM

^b^Standard deviation not reported

^c^ Assumption that oral tracer and dietary oxalate absorbed equally

### Various precursors of endogenous oxalate production

Seven studies investigated potential precursors of endogenous oxalate production and are summarized in Table 3. Four of those were excluded from pooled analysis due to high risk of bias as they did not control for exogenous oxalate consumption. The remaining studies found that hydroxyproline breakdown accounted for around 15% of endogenous oxalate production [19], glycine’s contribution was dose dependent but less than 5% at physiologic doses [17], phenylalanine’s contribution was < 0.7% (less than the lower limit of test detection) [17], and fructose’s contribution was negligible [21].

**Table 3:**
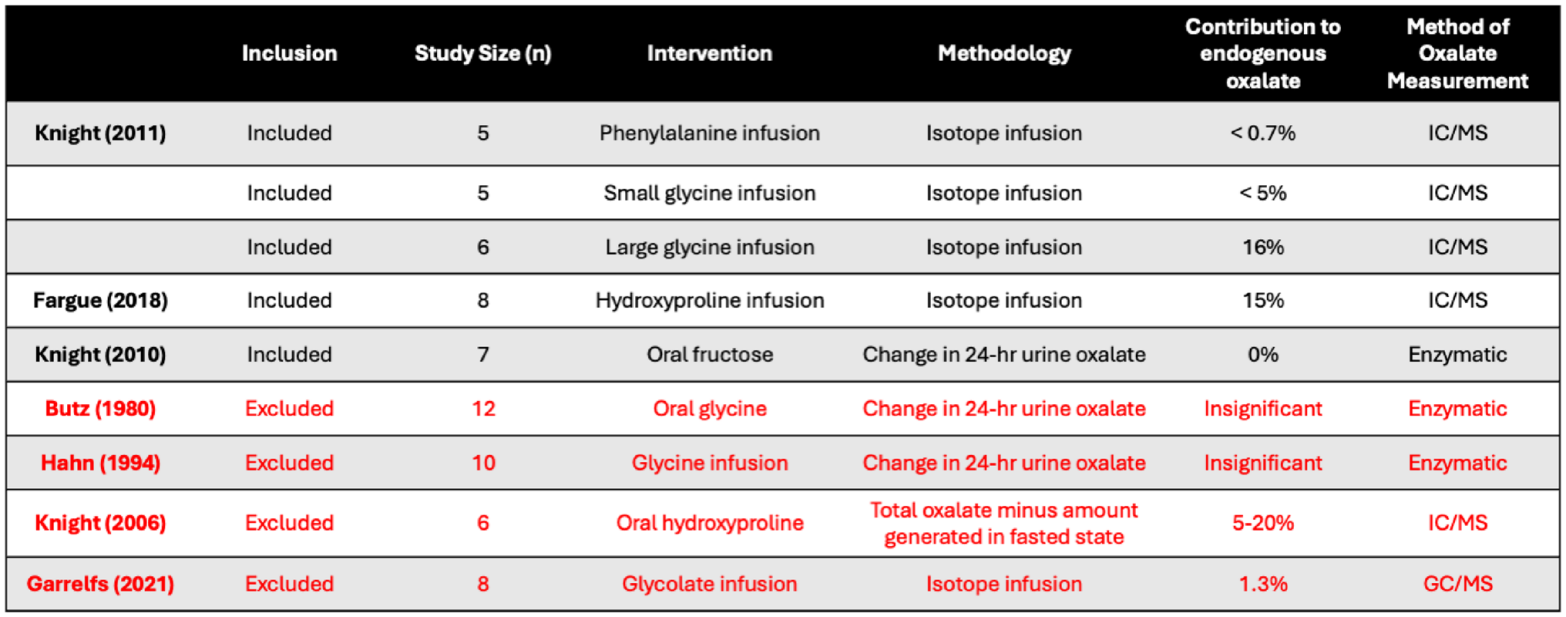
Studies investigating potential precursors of endogenous oxalate. GC/MS = gas chromatography and mass spectroscopy, IC/MS = ion chromatography and mass spectroscopy

### Ascorbic acid and endogenous oxalate production

Ten studies investigated the role of ascorbic acid as a precursor to oxalate (Table 4) yet only 3 had a low risk of bias. Butz et al. [15], Wandzilak et al. [24], Robitaille et al. [25], Baxmann et al. [27], Peña de la Vega et al. [28], and Hellman et al. [29] were excluded as they did not control for exogenous oxalate consumption. Urivetzky et al. [23] were excluded because they did not acidify patient urine. Many of these studies tested several different doses of ascorbic acid. Table 4 only includes the dose responsible for the highest conversion of ascorbic acid into oxalate. Tsao et al. [22] reported that supplementing ascorbic acid up to 10 g per day did not result in significant increases in oxalate excretion, however they did not provide any raw data. Chai et al. [10] also found no statistically significant change in oxalate excretion after supplementing 2 g/d ascorbic acid. Despite this, the group receiving ascorbic acid did have an extra ~4 mg of oxalate in their 24-hour urine samples, representing a 0.4% conversion. In contrast, Traxer et al. did observe a statistically significant mean increase of 6 mg / day (1.2% conversion) following 1 g/day supplementation with ascorbic acid [26].

**Table 4:**
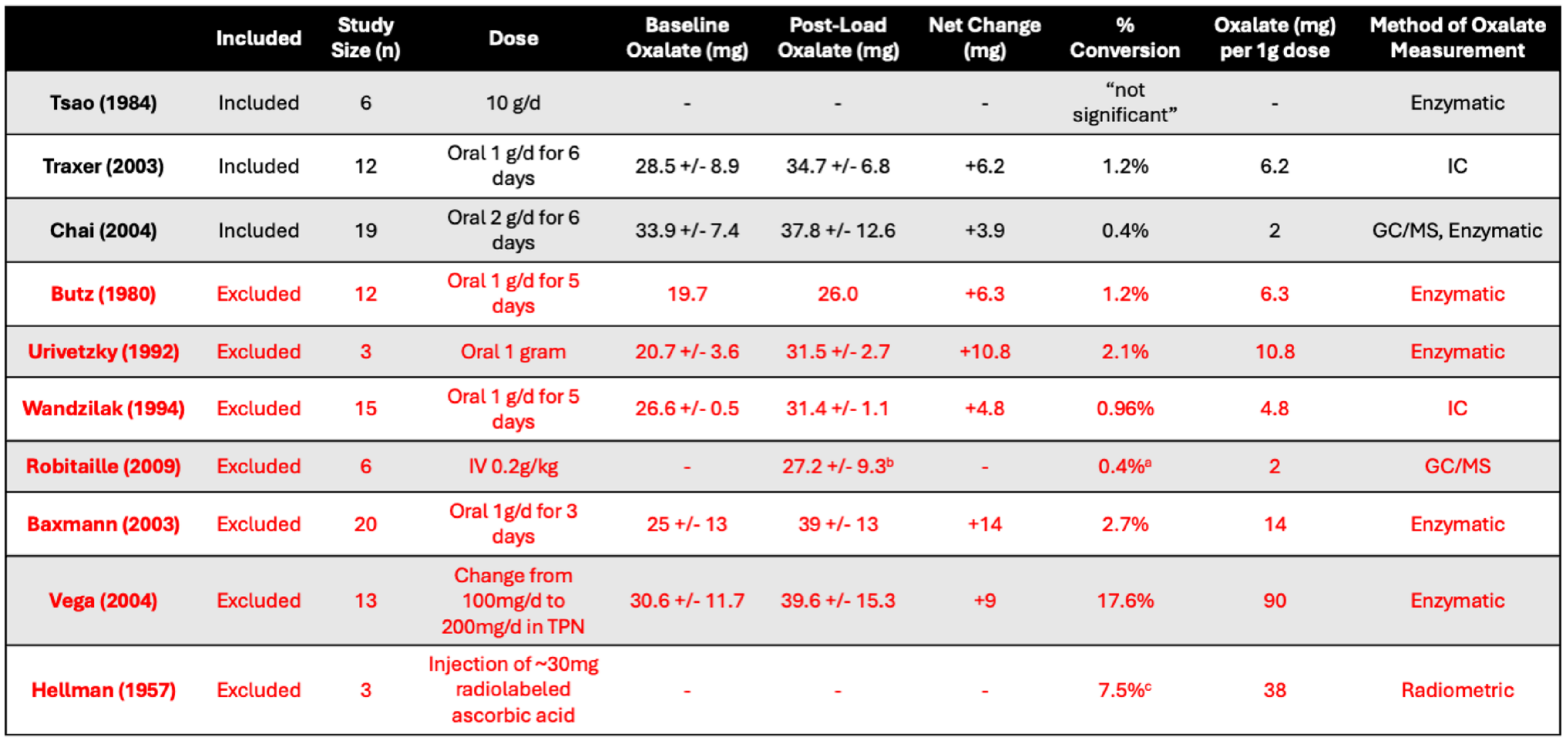
Studies investigating the role of ascorbic acid in endogenous oxalate synthesis.Baseline oxalate and post load oxalate determined from 24-hour urine collection unlessotherwise noted. GC/MS = gas chromatography and mass spectroscopy, IC/MS = ionchromatography and mass spectroscopy

^a^ Assuming all oxalate is from ascorbic acid

^b^ As determined at 6 h post-dose

^c^ As determined at 48-hour post-dose

Due to the limited number of studies, a funnel plot could not be performed to assess for reporting bias. Because most of the studies are observational, the evidence is low certainty.

## Discussion

**Fig. 2:**
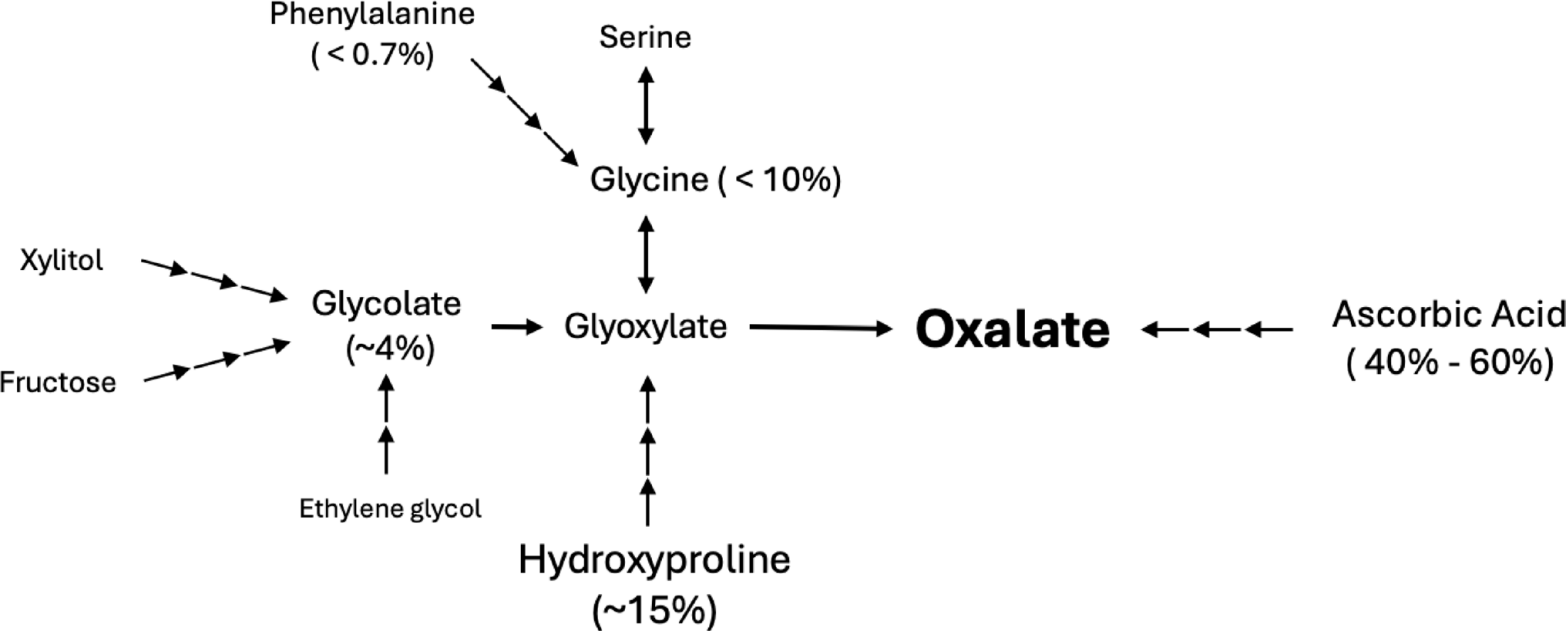
Sources of endogenous oxalate. Estimates of relative contribution to endogenous oxalate shown when known

Reducing urinary oxalate is a mainstay of kidney stone prevention. The American Urological Association guidelines recommend that patients with calcium oxalate stones limit intake of oxalate rich foods [5]. Yet exogenous oxalate consumption is only part of the picture. Even on a high oxalate, low calcium diet, exogenous consumption is reported to only account for half of total urinary oxalate [4]. Pooled analysis identified a mean daily endogenous production of about 24 mg oxalate per day. Reducing endogenous oxalate production may be an untapped therapeutic strategy for patients with stones. Knowing where endogenous oxalate comes from is a prerequisite for identifying a therapeutic target. Figure 2 summarizes pathways of endogenous oxalate production described in this review.

Historically, defining endogenous sources of oxalate has been hampered by imprecise methodology. Without controlling for diet, it is impossible to determine if changes in oxalate are due to a study intervention or dietary discrepancies. The most common reason for a study to be excluded from analysis was high risk of bias due to improper control for exogenous oxalate. Equally critical is acidification of urine after collection – a step that prevents calcium oxalate precipitation and non-enzymatic breakdown of ascorbic acid to oxalate [30]. Yet, even when controlling for diet and acidification of sample urine, other factors such as kidney function, obesity, and history of stones can predict abnormal oxalate handling and may confound results. Many of the studies stated their participants were “healthy” but did not explicitly rule out elevated BMI or creatinine, adding a layer of unknown heterogeneity.

This review identified hydroxyproline, glycine, phenylalanine, glycolate, fructose, and ascorbic acid as possible precursors to endogenous oxalate production. Many studies used intravenous infusion of isotopes to calculate overall flux and conversion to oxalate. Fargue et al. [19] performed a hydroxyproline isotope infusion and determined that it accounted for ~ 15% of endogenous oxalate [19]. They did not reach steady state in their experiment and thus may be underestimating hydroxyproline’s contribution. Phenylalanine [17] and fructose [21] contributed minimally to endogenous oxalate production.

Garrelfs et al. [20] gave participants an intravenous glycolate isotope and found that it accounted for 1.3% of endogenous oxalate production. However, this study was excluded from pooled analysis due to a high risk of bias from lack of dietary control. This lack of dietary restriction resulted in a total oxalate of ~ 70 mg, nearly 3 times the average amount calculated in this study. They reported that 0.9 mg/ day oxalate came from glycolate. Assuming a mean endogenous production of 24 mg, glycolate could account for up to 3.75%.

Glycine contribution appears to be dose dependent [17]. During a small volume infusion, dosed to determine glycine contribution in a fasted state, it accounted for less than 5% of endogenous oxalate production. During a larger volume infusion, dosed to determine glycine contribution after a high protein meal (i.e. 300 g roast beef), it accounted for 16% of endogenous oxalate production. The authors state that isotopic equilibrium was not reached during the larger infusion, suggesting that the *instantaneous* conversion rate during excess glycine may be higher. However, when considering a *24-hour period*, it is important to note that this 16% is likely a massive overestimate, as maintaining this state would require eating an equivalent of 300 g of roast beef every 4–5 h [31]. Therefore, even if the average person spends half their day in a fed, high protein state, glycine likely accounts for no more than 10% of endogenous oxalate.

Ascorbic acid’s role in endogenous oxalate synthesis has been controversial. While early studies suggested that up to 30% of ascorbic acid could be converted to oxalate, many argued that this was an artifact caused by improper handling of the urine samples [22, 32]. After exposure to oxygen in a collection container, ascorbic acid can undergo non-enzymatic conversion into oxalate [33]. This interference has been well-documented, necessitating strict urine handling procedures to prevent artifacts in measured oxalate levels [34]. To counteract this process, researchers now add acid to the urine collection jar. Urivetzky et al. [23] was the only study to not acidify their urine samples, instead adding EDTA and thimerosal. This method had questionable efficacy as they had one of the highest conversion rates (2.1%).

Of the included studies, Chai et al. [10] and Traxer et al. [26] provided enough data to calculate the percent conversion of ascorbic acid to oxalate. They respectively gave patients 2 g/ day and 1 g/ day and found that 0.4% and 1.2% of ascorbic acid was converted to oxalate, representing approximately 2 mg and 6 mg of oxalate per 1 g of supplemental ascorbic acid. Assuming an endogenous oxalate production of 24 mg/ day, this would account for between 8 and 25%. This wide range may be explained by the difference in dosing. If there is a limit to the amount of ascorbic acid that can convert to oxalate before it is filtered by the kidneys, larger doses would result in a lower percent conversion.

This saturation hypothesis is further supported by Robitaille et al. [25]. Although this study was excluded from primary analysis due to lack of dietary control, certain study arms should be less affected by dietary bias than others. Dietary differences may confound effects of small treatment doses, but very large treatment doses should outweigh any background noise. In the highest dose group, Robitaille et al. [25] gave participants a supraphysiologic, intravenous dose of 1.5 g/kg and found that merely 0.2% was converted to oxalate. This supports the theory that *supraphysiologic doses* of ascorbic acid play a limited role in endogenous oxalate production. However, at *physiologic doses*, this may not hold true.

The Hellman et al. [29] study was excluded from pooled analysis because it did not control for exogenous oxalate consumption. However, by using a radiolabeled ascorbic acid tracer, the authors were able to track its conversion into oxalate irrespective of diet. In this 1957 study, vitamin-replete subjects received a small oral tracer dose of ascorbic acid (24-38 mg) and daily urinary excretion of radiolabeled ascorbic acid and oxalate was measured. Flux equations were used to estimate the metabolic pool of ascorbic acid (~ 20 mg/kg), the half-life (~ 16 days), the daily turnover (~ 1 mg /kg /day), and the percent of the ascorbic acid that becomes urinary oxalate (~ 40%). For a 70 kg adult, this translates to roughly 14 mg of oxalate generated from daily turnover from the metabolic pool of ascorbic. Assuming a mean endogenous oxalate of 24 mg, this represents nearly 60% of endogenously produced oxalate. However, caution must be taken when interpreting these mean values as they mask substantial inter-subject variability.

**Fig. 3:**
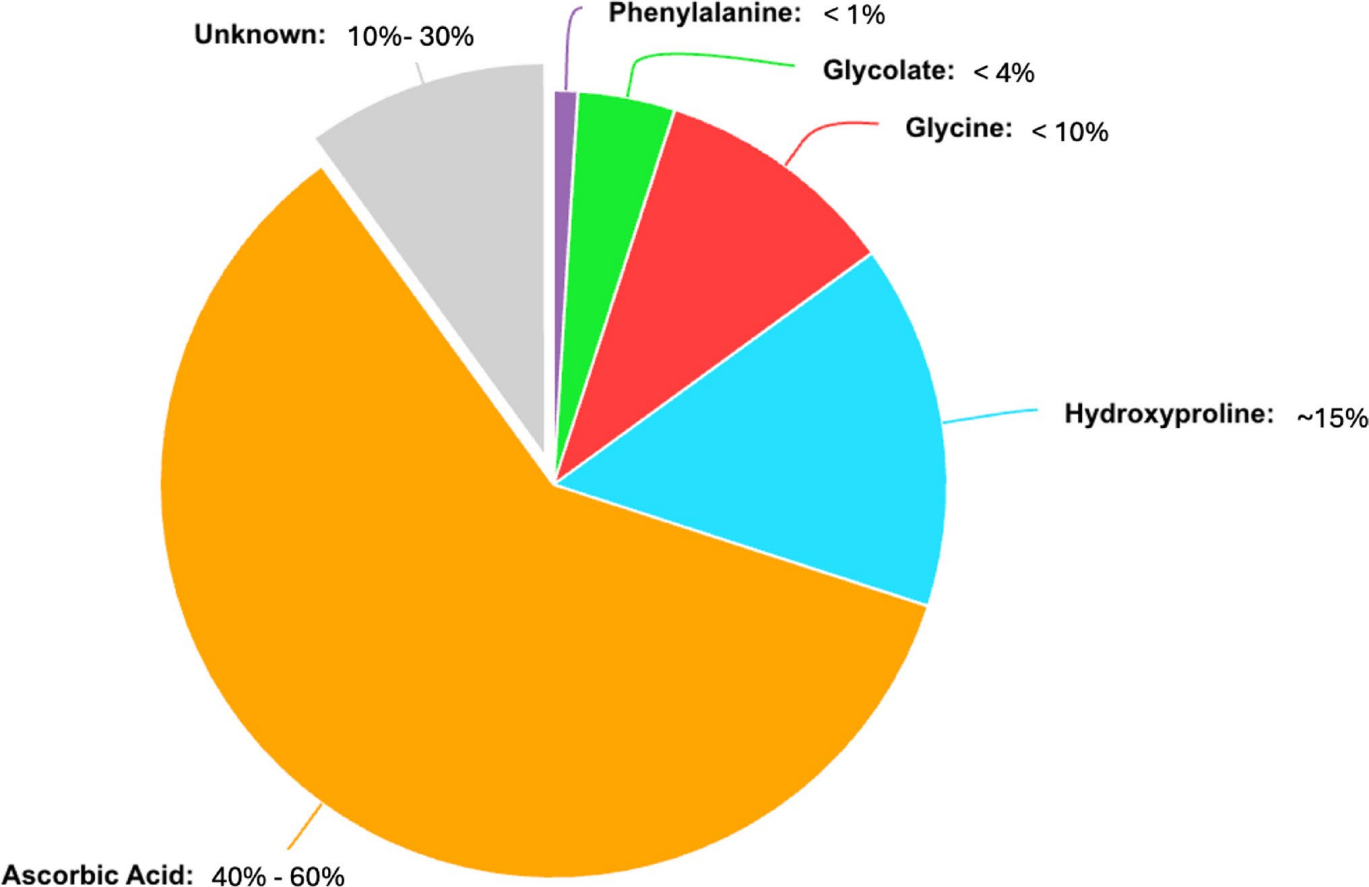
Tentative representation of contribution of precursors to endogenous oxalate production

Analyzing individual data from Hellman et al. [29] reveals that daily turnover from the metabolic pool of ascorbic acid ranged two-fold, from 0.66 to 1.4 mg /kg /day. Additionally, the percent of radiotracer excreted as oxalate in the urine ranged 33% − 59%. Consequently, in a 70 kg individual, the calculated daily oxalate burden from daily turnover from the metabolic pool of ascorbic acid could range from as low as 7.7 mg to as high as 21 mg. Comparisons with other radiotracer studies confirm this broad range. Atkins et al. [35] and Abt et al. [36] administered oral tracers to participants and observed the turnover rate from the metabolic pool of ascorbic acid to be 1.25 mg /kg /day and 0.4 mg /kg /day respectively. Thus, while turnover from the metabolic pool of ascorbic acid appears to be the major precursor of endogenous oxalate, future studies are needed to define the magnitude of its effect. Figure 3 shows each precursor discussed in this review, as well as their relative contribution to endogenous oxalate.

Defining an exact physiological threshold at which ascorbic acid confers a definitive lithogenic risk remains elusive. Clinically, it is necessary to distinguish between baseline metabolic requirements and the intake of supraphysiologic supplements. Ascorbic acid utilized for normal daily function enters the metabolic pool, allowing a substantial proportion to undergo conversion into oxalate over several weeks. In contrast, excess intake saturates tissue stores and is rapidly cleared by the kidneys. While the majority of supraphysiologic ascorbic acid is excreted before it can be metabolized, modern evidence demonstrates that a 1 g dose can still cause a clinically relevant increase in absolute urinary oxalate excretion [10, 26]. As lower absolute urinary oxalate excretion is independently associated with decreased relative risk of stone formation, any unnecessary increase poses a lithogenic risk [3]. Consequently, high dose ascorbic acid supplementation should be avoided when possible in patients with recurrent calcium oxalate stones.

There are several limitations to this review. First, there is inherent subjectivity of assessing study bias. To mitigate this, the modified JBI checklist tool and critical domains were established prior to data extraction. Second, the search strategy was designed to identify primary literature that used metabolic study methodology. While this prioritized accuracy and precision of findings, it inherently limited the scope to a smaller number of studies, potentially excluding larger observational data. Accordingly, many of the findings of this paper come from single studies. It must also be emphasized that the radiotracer studies detailing the daily turnover from the metabolic pool of ascorbic acid relied on small sample sizes (*N* = 1 to 4) and are now nearly 70 years old. Additionally, the Hellman radiotracer study [29] derived its estimates from three hospitalized patients suffering from systemic illnesses, including multiple sclerosis and metastatic breast cancer. Applying these 1950s metabolic estimates from chronically ill, hospitalized patients to modern, healthy individuals introduces a significant demographic mismatch that severely limits generalizability. The estimate that 40% to 60% of total endogenous oxalate comes from turnover of the metabolic pool of ascorbic acid should be interpreted with caution. Lastly, estimates of endogenous oxalate production can be confounded by the method used to determine exogenous oxalate absorption. Many studies [8–10, 12] used oral oxalate isotope absorption to estimate exogenous oxalate absorption. Zimmermann et al. [11] note that this supplemental oxalate is likely absorbed more readily than dietary oxalate, causing studies to overestimate exogenous absorption and underestimate endogenous oxalate production.

Despite these limitations, the strength of this review comes from its inclusion of scientifically rigorous metabolic data. Through compilation of these studies, this review constructs a metabolic map of endogenous oxalate production, delineating the relative contributions of known precursors. Through this synthesis, a critical knowledge gap has been identified – the reliance on historical, highly variable data regarding turnover from the metabolic pool of ascorbic acid. Future studies must reinvestigate this pathway using modern methodology and technology.

## Conclusion

Endogenous sources of oxalate include ascorbic acid and amino acids like glycine and hydroxyproline. While supraphysiologic doses of ascorbic acid are filtered by the kidneys prior to significant conversion to oxalate, daily turnover from the metabolic pool of ascorbic acid may account for up to 40% − 60% of total endogenous oxalate production. However, studies defining this contribution are now half a century old and suffer from inter-study and intra-study variability. Future work must utilize modern metabolic techniques to definitively establish ascorbic acid’s role in oxalate metabolism. In practice, high-dose ascorbic acid supplementation yields clinically relevant increases in absolute urinary oxalate and should be avoided when possible in patients with recurrent calcium oxalate stones.

## Supplementary Information

Below is the link to the electronic supplementary material.

Supplementary Material 1

## Data Availability

All data generated or analyzed during this study are included in this published article and its supplementary information files.
