# Supplementary material for: Where does endogenous oxalate come from? - a systematic review of endogenous sources of urinary oxalate
Source: Urolithiasis. 2026 Apr 30;54(1):98. doi: 10.1007/s00240-026-01995-2 (PMC13133231; doi:10.1007/s00240-026-01995-2)
Supplement: Supplementary file 1 — Supplementary Material 1 [file 240_2026_1995_MOESM1_ESM.docx]

**Supplementary Files**

**S1. Search string used for PubMed and Embase**

**S2. Bias assessment tool**

**S3. PRISMA Checklist**

**S1.** Database search strings

**PubMed Search String:**

(oxalates[MeSH Major Topic] OR hyperoxaluria[MeSH Major Topic])

AND

(urine[MeSH Major Topic] OR urinary[Title/Abstract] OR urine[Title/Abstract])

AND

(endogenous[Title/Abstract] OR synthesis[Title/Abstract] OR production[Title/Abstract] OR precursor[Title/Abstract] OR glycolate[Title/Abstract] OR glyoxylate[Title/Abstract] OR (ascorbic acid[Title/Abstract]) OR (vitamin C[Title/Abstract]) OR hydroxyproline[Title/Abstract])

AND

((mass spectrometry) OR (spectrophotometric) OR (fluorescence) OR (chromatography) OR (GC-MS) OR (HPLC) OR (LC-MS) OR (LC-MS/MS) OR (infusion) OR (tracer) OR (isotope) OR (labeled) OR (radiolabeled) OR (13C) OR (14C) OR (isotope dilution) OR (load) OR (loading) OR (kinetics) OR (quantification) OR (gold standard) OR (enzymatic) OR (hourly urinary oxalate))

AND

(English[Language])

**Embase Search String:**

('oxalic acid'/exp/mj OR 'hyperoxaluria'/exp/mj)

AND

('urine'/exp/mj OR urine:ti,ab OR urinary:ti,ab)

AND

(endogenous:ti,ab OR synthesis:ti,ab OR production:ti,ab OR precursor:ti,ab OR glycolate:ti,ab OR glyoxylate:ti,ab OR 'ascorbic acid':ti,ab OR 'vitamin c':ti,ab OR hydroxyproline:ti,ab)

AND

('mass spectrometry':ti,ab OR spectrophotometric:ti,ab OR fluorescence:ti,ab OR chromatography:ti,ab OR 'gc-ms':ti,ab OR hplc:ti,ab OR 'lc-ms':ti,ab OR 'lc-ms/ms':ti,ab OR infusion:ti,ab OR tracer:ti,ab OR isotope:ti,ab OR labeled:ti,ab OR radiolabeled:ti,ab OR '13c':ti,ab OR '14c':ti,ab OR 'isotope dilution':ti,ab OR load:ti,ab OR loading:ti,ab OR kinetics:ti,ab OR quantification:ti,ab OR 'gold standard':ti,ab OR enzymatic:ti,ab OR 'hourly urinary oxalate':ti,ab)

AND

[english]/lim

**S2. Bias appraisal tool**

**JBI Critical Appraisal Checklist: Modified Case Series**

Author_______________________________________ Year_________ Record Number_________

|  | Yes | No | Unclear | Not applicable |
| --- | --- | --- | --- | --- |
| Does the study involve participants that were free of genetic or renal defects that alter oxalate handling (i.e. CKD or primary hyperoxaluria)? | □ | □ | □ | □ |
| Does the study outline patient demographics possibly related to endogenous oxalate synthesis (i.e. BMI, stone formers and non-stone formers)? | □ | □ | □ | □ |
| Does the study control for diet/ exogenous oxalate consumption? | □ | □ | □ | □ |
| If an oral tracer was used, did the study account for the higher bioavailability of the tracer relative to dietary oxalate? | □ | □ | □ | □ |
| Is urine acidified upon collection to prevent non-enzymatic conversion of ascorbic acid to oxalate? | □ | □ | □ | □ |
| Was the oxalate assayed in a reliable way for all participants? | □ | □ | □ | □ |
| For tracer studies, was steady state reached? | □ | □ | □ | □ |

Overall appraisal: Include □ Exclude □ Seek further info □

Comments (Including reason for exclusion)

_____________________________________________________________________________________________

_____________________________________________________________________________________________

**S3. PRISMA Checklist**

| **Section and Topic** | **Item #** | **Checklist item** | | | **Location where item is reported** |
| --- | --- | --- | --- | --- | --- |
| **TITLE** | | |  |  |  |
| Title | 1 | Identify the report as a systematic review. | | | Title |
| **ABSTRACT** | | |  |  |  |
| Abstract | 2 | See the PRISMA 2020 for Abstracts checklist. | | | Abstract |
| **INTRODUCTION** | | |  |  |  |
| Rationale | 3 | Describe the rationale for the review in the context of existing knowledge. | | | Paragraph 2 |
| Objectives | 4 | Provide an explicit statement of the objective(s) or question(s) the review addresses. | | | Paragraph 3 |
| **METHODS** | | |  |  |  |
| Eligibility criteria | 5 | Specify the inclusion and exclusion criteria for the review and how studies were grouped for the syntheses. | | | *Search string* |
| Information sources | 6 | Specify all databases, registers, websites, organisations, reference lists and other sources searched or consulted to identify studies. Specify the date when each source was last searched or consulted. | | | *Search string* |
| Search strategy | 7 | Present the full search strategies for all databases, registers and websites, including any filters and limits used. | | | Supplemental file |
| Selection process | 8 | Specify the methods used to decide whether a study met the inclusion criteria of the review, including how many reviewers screened each record and each report retrieved, whether they worked independently, and if applicable, details of automation tools used in the process. | | | *Article review* |
| Data collection process | 9 | Specify the methods used to collect data from reports, including how many reviewers collected data from each report, whether they worked independently, any processes for obtaining or confirming data from study investigators, and if applicable, details of automation tools used in the process. | | | *Data extraction and analysis* |
| Data items | 10a | List and define all outcomes for which data were sought. Specify whether all results that were compatible with each outcome domain in each study were sought (e.g. for all measures, time points, analyses), and if not, the methods used to decide which results to collect. | | | *Data extraction and analysis* |
|  | 10b | List and define all other variables for which data were sought (e.g. participant and intervention characteristics, funding sources). Describe any assumptions made about any missing or unclear information. | | | *Data extraction and analysis* |
| Study risk of bias assessment | 11 | Specify the methods used to assess risk of bias in the included studies, including details of the tool(s) used, how many reviewers assessed each study and whether they worked independently, and if applicable, details of automation tools used in the process. | | | *Bias assessment* |
| Effect measures | 12 | Specify for each outcome the effect measure(s) (e.g. risk ratio, mean difference) used in the synthesis or presentation of results. | | | *Data extraction and analysis* |
| Synthesis methods | 13a | Describe the processes used to decide which studies were eligible for each synthesis (e.g. tabulating the study intervention characteristics and comparing against the planned groups for each synthesis (item #5)). | | | *Data extraction and analysis* |
|  | 13b | Describe any methods required to prepare the data for presentation or synthesis, such as handling of missing summary statistics, or data conversions. | | | *Data extraction and analysis* |
|  | 13c | Describe any methods used to tabulate or visually display results of individual studies and syntheses. | | | *Data extraction and analysis* |
|  | 13d | Describe any methods used to synthesize results and provide a rationale for the choice(s). If meta-analysis was performed, describe the model(s), method(s) to identify the presence and extent of statistical heterogeneity, and software package(s) used. | | | *Data extraction and analysis* |
|  | 13e | Describe any methods used to explore possible causes of heterogeneity among study results (e.g. subgroup analysis, meta-regression). | | | N/A |
|  | 13f | Describe any sensitivity analyses conducted to assess robustness of the synthesized results. | | | N/A |
| Reporting bias assessment | 14 | Describe any methods used to assess risk of bias due to missing results in a synthesis (arising from reporting biases). | | | N/A |
| Certainty assessment | 15 | Describe any methods used to assess certainty (or confidence) in the body of evidence for an outcome. | | | N/A |
| **RESULTS** | | |  |  |  |
| Study selection | 16a | Describe the results of the search and selection process, from the number of records identified in the search to the number of studies included in the review, ideally using a flow diagram. | | | Results paragraph 1 |
|  | 16b | Cite studies that might appear to meet the inclusion criteria, but which were excluded, and explain why they were excluded. | | | N/A |
| Study characteristics | 17 | Cite each included study and present its characteristics. | | | Split between paragraph 1 of results and the table of the results |
| Risk of bias in studies | 18 | Present assessments of risk of bias for each included study. | | | *Risk of bias* in results, Table 1 |
| Results of individual studies | 19 | For all outcomes, present, for each study: (a) summary statistics for each group (where appropriate) and (b) an effect estimate and its precision (e.g. confidence/credible interval), ideally using structured tables or plots. | | | Table 2-4 |
| Results of syntheses | 20a | For each synthesis, briefly summarise the characteristics and risk of bias among contributing studies. | | | Table 1 |
|  | 20b | Present results of all statistical syntheses conducted. If meta-analysis was done, present for each the summary estimate and its precision (e.g. confidence/credible interval) and measures of statistical heterogeneity. If comparing groups, describe the direction of the effect. | | | Table 2-4 |
|  | 20c | Present results of all investigations of possible causes of heterogeneity among study results. | | | N/A |
|  | 20d | Present results of all sensitivity analyses conducted to assess the robustness of the synthesized results. | | | N/A |
| Reporting biases | 21 | Present assessments of risk of bias due to missing results (arising from reporting biases) for each synthesis assessed. | | | Limitations (2^nd^ to last paragraph of discussion) |
| Certainty of evidence | 22 | Present assessments of certainty (or confidence) in the body of evidence for each outcome assessed. | | | N/A |
| **DISCUSSION** | | |  |  |  |
| Discussion | 23a | Provide a general interpretation of the results in the context of other evidence. | | | Throughout discussion |
|  | 23b | Discuss any limitations of the evidence included in the review. | | | Limitations (2^nd^ to last paragraph of discussion) |
|  | 23c | Discuss any limitations of the review processes used. | | | Limitations (2^nd^ to last paragraph of discussion) |
|  | 23d | Discuss implications of the results for practice, policy, and future research. | | | Last paragraph of discussion |
| **OTHER INFORMATION** | | |  |  |  |
| Registration and protocol | 24a | Provide registration information for the review, including register name and registration number, or state that the review was not registered. | | | Not registered, discussed in method. The inclusion criteria and search strategy were established prior to data extraction; however we failed to register the study online prior to data extraction. |
|  | 24b | Indicate where the review protocol can be accessed, or state that a protocol was not prepared. | | | Not registered, discussed in method. The inclusion criteria and search strategy were established prior to data extraction; however we failed to register the study online prior to data extraction. |
|  | 24c | Describe and explain any amendments to information provided at registration or in the protocol. | | | Not registered, discussed in method |
| Support | 25 | Describe sources of financial or non-financial support for the review, and the role of the funders or sponsors in the review. | | | No disclosures, mentioned in declaration |
| Competing interests | 26 | Declare any competing interests of review authors. | | | No competing interests |
| Availability of data, code and other materials | 27 | Report which of the following are publicly available and where they can be found: template data collection forms; data extracted from included studies; data used for all analyses; analytic code; any other materials used in the review. | | | All data available in paper and supplemental files |

*From:*  Page MJ, McKenzie JE, Bossuyt PM, Boutron I, Hoffmann TC, Mulrow CD, et al. The PRISMA 2020 statement: an updated guideline for reporting systematic reviews. BMJ 2021;372:n71. doi: 10.1136/bmj.n71. This work is licensed under CC BY 4.0. To view a copy of this license, visit <https://creativecommons.org/licenses/by/4.0/>
